# Burden, trends, and risk factors of esophageal cancer in China from 1990 to 2017: an up-to-date overview and comparison with those in Japan and South Korea

**DOI:** 10.1186/s13045-020-00981-4

**Published:** 2020-11-02

**Authors:** Si Yang, Shuai Lin, Na Li, Yujiao Deng, Meng Wang, Dong Xiang, Grace Xiang, Shuqian Wang, Xianghua Ye, Yi Zheng, Jia Yao, Zhen Zhai, Ying Wu, Jingjing Hu, Huafeng Kang, Zhijun Dai

**Affiliations:** 1 Department of Breast Surgery, The First Affiliated Hospital, School of Medicine, Zhejiang University, Hangzhou, 310003 Zhejiang China; 2 Department of Oncology, The Second Affiliated Hospital of Xi’an Jiaotong University, Xi’an, 710004 Shaanxi China; 3 Celilo Cancer Center, Oregon Health Science Center Affiliated Mid-Columbia Medical Center, The Dalles, OR USA; 4 College of Arts and Sciences, New York University, New York, NY USA; 5 Department of Radiotherapy, The First Affiliated Hospital, School of Medicine, Zhejiang University, Hangzhou, 310003 Zhejiang China; 6 Dana-Farber Cancer Institute, Harvard Medical School, Boston, MA USA

**Keywords:** Death, Disability-adjusted life years, Esophageal cancer, Incidence, Risk factor

## Abstract

**Background:**

The epidemiology of esophageal cancer (EC) can elucidate its causes and risk factors and help develop prevention strategies. We aimed to provide an overview of the burden, trends, and risk factors of EC in China from 1990 to 2017. We also investigated the differences between China, Japan, and South Korea and discussed the possible causes of the disparities.

**Methods:**

We used the Global Burden of Disease Study 2017 to obtain data on incident cases, deaths, disability-adjusted life-year (DALY) cases, age-standardized incidence rate (ASIR), age-standardized death rate (ASDR), and age-standardized DALY rate of EC in China, Japan, and South Korea from 1990 to 2017. Trend analysis was performed using joinpoint analysis. We measured the associations between ASIR, ASDR, and age-standardized DALY rate and the socio-demographic index (SDI) for 1990–2017. We also analyzed the risk factors associated with EC deaths and DALYs.

**Results:**

China recorded 234,624 (95% uncertainty intervals: 223,240–246,036) incident cases of and 212,586 (202,673–222,654) deaths from EC in 2017. The ASIR and ASDR declined from 1990 to 2017. Until 2017, the ASIR was 12.23, and ASDR was 11.25 per 100,000 persons. The DALYs were 4,464,980 (4,247,816–4,690,846) with an age-standardized rate of 222.58 per 100,000 persons in 2017. The ASIR, ASDR, and age-standardized DALY rate in China were twice those of Japan and South Korea. These three indicators showed a decreasing trend, whereas SDI increased, in all three countries from 1990 to 2017. Tobacco and alcohol use remained the major risk factors for EC death and DALYs, especially for men in China and women in Japan and South Korea. High body mass index (BMI) and low-fruit diet were the main risk factors for women in China.

**Conclusions:**

The incident cases and deaths of EC in China, Japan, and South Korea increased from 1990 to 2017, whereas the ASIR, ASDR, and age-standardized DALY rate declined. China had the greatest burden of EC among three countries. SDI and aging along with tobacco use, alcohol use, high BMI, and low-fruit diet were the main risk factors of death and DALYs and should be paid more attention.

## Background

Esophageal cancer (EC), the seventh most common cancer and the sixth leading cause of cancer death worldwide [1], etiologically includes two main histological subtypes: esophageal squamous cell carcinoma (ESCC, about 90%) and adenocarcinoma (EAC, about 10%) [2]. EC poses a bigger threat to less developed than developed regions [3] and is a complex disease with various causes, although alcohol and tobacco are well-known causes [4, 5]. Higher consumption of vegetables and fruits and increased physical activity are correlated with a low risk of EC [6], whereas intake of pickled vegetables is correlated with a high risk [7]. Smoking, alcohol use, and low intake of fruits and vegetables are reported to account for 89% of the risk factors of EC in the USA [6]. Larger samples of EC are needed to explore these risk factors, which have changed remarkably over the decades. The incidence and mortality trends of EC worldwide have also changed markedly, with huge heterogeneity between sex, histological type, and geographical distribution. For example, in the USA and some other Western countries, the incidence of EAC has increased and is the major type of EC, whereas that of ESCC has been declining in the past 30 years [8]. The East Asian countries of China, Japan, and South Korea carry a heavy burden of EC; ESCC is the predominant type, whereas EAC remains rare [9–12]. Although possessing similar racial, genetic, and cultural backgrounds, China, Japan, and South Korea are at different stages of socioeconomic development. The economic growth period of China occurred later than that of Japan and South Korea. The nationwide cancer control programs of China were also implemented later [13, 14]. The comparison of the burden, trends, and risk factors of EC in these three East Asian countries could be useful to track the effectiveness of national screening programs and provide a scientific reference for cancer control policy planning in China.

The current study aimed to provide an up-to-date overview of the burden, trends, and risk factors of EC in China from 1990 to 2017, compared with Japan and South Korea, and to discuss the potential reasons for the disparities. In considering the availability and comparability of data, we conducted our analysis using data from the Global Burden of Disease Study 2017. We investigated the factors for EC, including the socio-demographic index (SDI), age, sex, and various risk exposures, to contribute to strategies for health promotion.

## Methods

### Data source

We obtained data on incident cases, deaths, disability-adjusted life-years (DALYs), age-standardized incidence rate (ASIR), age-standardized death rate (ASDR), and age-standardized DALY rate of EC in the world, East Asia and Pacific region, China, Japan, and South Korea for the period of 1990–2017 from the Global Burden of Disease Study 2017 [15, 16]. The East Asia and Pacific region mainly includes China, Japan, Mongolia, Singapore, and North and South Korea in the present study. We extracted data using the Global Health Data Exchange query tool (https://ghdx.healthdata.org/gbd-results-tool). In the case of China, we analyzed data on a total of 34 province-level administrative units, including 22 provinces, four municipalities, five autonomous regions, Taiwan, Hong Kong Special Administrative Region (SAR), and Macao SAR. All of these are referred to as provinces throughout the present paper. According to the division of the National Bureau of Statistics of China, we classified the 31 provinces in mainland China into three areas according to geography: Eastern (*n* = 11), Middle (*n* = 8), and Western (*n* = 12). The DALYs and age-standardized DALY rate in 34 provinces of China from 1990 to 2017 were collected from a previous study [17]. The incident cases, death cases, ASIR, and ASDR for EC in three areas of China in 2015 were collected from the Chinese Cancer Registry Annual Report 2018. The SDI is a composite indicator of income per capita, average educational attainment, and total fertility rates; the index ranges from 0 to 1 [16]. In 2017, the SDI for China was 0.71, although it ranged from 0.47 to 0.86 at the provincial level. Thus, on a global scale, China is in the high-middle SDI quintile, whereas its 34 provinces vary from the low-middle to high SDI quintiles.

### Joinpoint regression analysis

We performed joinpoint regression analysis to determine the annual percent changes in the ASIR, ASDR, and age-standardized DALY rate of EC from 1990 to 2017 in the world, East Asia and Pacific, China, Japan, and South Korea using Joinpoint (version 4.7.0). Joinpoint regression analysis uses a piecewise linear regression method to determine the trend displayed by one or more line segments [18].

### Risk factor analysis

The exposure variables were divided into four large categories: environmental and occupational, behavioral, metabolic, and dietary risks. These risks are defined and described in the comparative risk assessment framework of the Global Burden of Disease Study 2017 [19].

### Statistical analyses

The standardized methods of the Global Burden of Disease Study 2017 have been reported [15]. To eliminate the effect of age composition from comparisons across various geographical regions or time periods, we used the ASIR, ASDR, and age-standardized DALY rate (per 100,000 populations) [20]. We generated the 95% uncertainty intervals (UI) for all reported data. All statistical analyses were performed using R software (version 3.5.2). A *p* value of < 0.05 was considered statistically significant.

## Results

### EC burden in the world, East Asia and Pacific, China, Japan, and South Korea

Table 1 shows the age-standardized rate (ASR) and number of EC cases in the world, East Asia and Pacific, China, Japan, and South Korea from 1990 to 2017. China had 234,624 all-age incident cases of EC (95% UI: 223,240–246,036), with an ASIR of 12.23 (95% UI: 11.64–12.82) per 100,000 persons in 2017. In the same year, China had 212,586 (95% UI: 202,673–222,654) deaths from EC, with an ASDR of 11.25 (95% UI: 10.73–11.77) per 100,000 persons. Meanwhile, the all-age DALYs were 4,464,980 (95% UI: 4,247,816–4,690,846), with an ASR of 222.58 (95% UI: 211.95–233.57) per 100,000 persons. The incident cases, deaths, and DALYs of EC in China accounted for 84.07%, 85.58%, and 84.84%, respectively, of EC burden in East Asia and Pacific and 49.65%, 48.76%, and 45.66%, respectively, of the global burden. Notably, the geographic distribution of EC varied greatly. The ASIR, ASDR, and age-standardized DALY rate of EC in China were about 1.3 times that of East Asia and Pacific, twice that of the world, and more than twice that of Japan and South Korea (Table 1).

**Table 1:** The age-standardized rate and number of esophageal cancer cases in the world, East Asia and Pacific, China, Japan, and South Korea from 1990 to 2017, both sexes

Variables	World			East Asia and Pacific			China			Japan			South Korea		
	1990	2017	Change, %	1990	2017	Change, %	1990	2017	Change, %	1990	2017	Change, %	1990	2017	Change, %
Incidence rate (per 100,000)	7.57 (7.33–7.85)	5.90 (5.74–6.06)	− 22.06	13.78 (13.22–14.50)	9.22 (8.84–9.60)	− 33.07	19.38 (18.52–20.50)	12.23 (11.64–12.82)	− 36.89	6.09 (5.99–6.19)	5.83 (5.52–6.13)	− 4.27	5.04 (4.84–5.26)	3.25 (2.81–3.72)	− 35.52
Incidence numbers	310,236 (300,690–322,028)	472,525 (459,485–485,294)	52.31	187,945 (180,252–197,677)	279,072 (267,563–290,531)	48.49	164,473 (157,194–173,853)	234,624 (223,240–246,036)	42.65	10,667 (10,485–10,848)	19,202 (18,203–20,140)	80.01	1,608 (1,541–1,679)	2,801 (2,414–3,216)	74.19
Death rate (per 100,000)	7.72 (7.48–8.01)	5.48 (5.34–5.63)	− 29.02	14.23 (13.63–15.00)	8.27 (7.95–8.61)	− 41.85	20.53 (19.60–21.71)	11.25 (10.73–11.77)	− 45.20	4.47 (4.41–4.53)	3.66 (3.51–3.81)	− 18.12	5.12 (4.92–5.32)	2.16 (1.92–2.41)	− 57.81
Death numbers	311,289 (301,451–323,235)	435,959 (424,994–447,580)	40.05	188,862 (180,759–198,909)	248,410 (238,407–258,346)	31.53	168,455 (160,933–178,231)	212,586 (202,673–222,654)	26.20	7,780 (7,673–7,897)	12,807 (12,310–13,311)	64.61	1,577 (1,515–1,644)	1,865 (1,653–2,078)	18.26
DALY rate (per 100,000)	179.94 (174.09–186.82)	119.91 (116.89–122.99)	− 33.36	314.57 (301.10–331.18)	169.37 (162.32–176.65)	− 46.16	446.42 (425.73–471.70)	222.58 (211.95–233.57)	− 50.14	96.88 (95.43–98.44)	77.78 (74.47–81.14)	− 19.72	124.46 (119.44–129.78)	44.57 (39.34–50.04)	− 64.19
DALY numbers	7,677,078 (7,422,100–7,970,949)	9,777,771 (9,532,791–10,031,497)	27.36	4,582,723 (4,386,849–4,832,515)	5,262,866 (5,042,535–5,489,756)	14.84	4,078,281 (3,890,143–4,314,430)	4,464,980 (4,247,816–4,690,846)	9.48	172,127 (169,542–174,893)	227,447 (218,017–236,700)	32.14	42,870 (41,041–44,820)	38,988 (34,415–43,822)	− 9.06

*DALY* disability-adjusted life-year

**Fig. 1:**
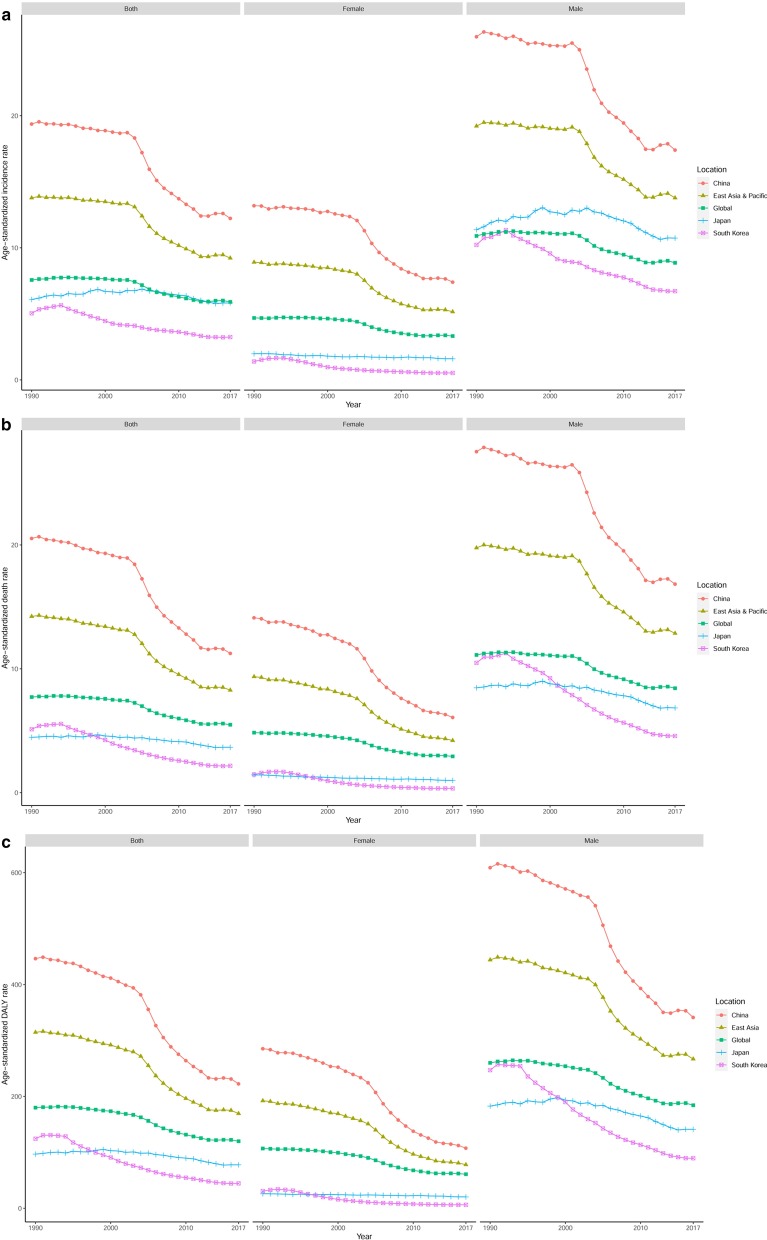
Trends for the ASIR (**a**), ASDR (**b**), and age-standardized DALY rate (**c**) of esophageal cancer in the world, East Asia and Pacific, China, Japan, and South Korea from 1990 to 2017. *ASIR* age-standardized incidence rate, *ASDR* age-standardized death rate, *DALY* disability-adjusted life-year

We observed an increasing trend in incident cases and deaths in the world, East Asia and Pacific, China, Japan, and South Korea for both sexes between 1990 and 2017 (Table 1). Among the three countries, the greatest increases were found in Japan (80.01% and 64.61%, respectively) and the lowest in China for incident cases and South Korea for deaths (42.65% and 18.26%, respectively). The ASIR, ASDR, and age-standardized DALY rate showed a decreasing trend in the world, East Asia and Pacific, and the three countries for both sexes between 1990 and 2017 (Table 1, Fig. 1). Among the three countries, the ASIR declined the most in China (− 36.89%) and the least in Japan (− 4.27%). The ASDR declined the most in South Korea (− 57.81%) and declined the least in Japan (− 18.12%). The age-standardized DALY rate declined the most in South Korea (− 64.19%) and declined the least in Japan (− 19.72%).

### Sex and age distribution pattern in the world, East Asia and Pacific, China, Japan, and South Korea

In 2017, 70.03% (330,904 [95% UI: 319,159–341,987]) of the incident cases occurred in men, compared with 29.97% (141,621[95% UI: 135,089–147,968]) in women worldwide (Table 2). The ASIR in men was about 1.7 times higher than that in women. In the same year, 71.3% (310,733 [95% UI: 300,244–320,551]) of the global deaths occurred in men, compared with 28.7% (125,226 [95%UI: 194 120,125–130,421]) in women. The ASDR in men was about 1.9 times higher than that in women. In 2017, 70.63% (197,115[95% UI: 186,163–207,760]) of the incident cases occurred in men, compared with 29.37% (81,957[95% UI: 76,083–87,598]) in women in East Asia and Pacific (Table 2). Moreover, the ASIR in men was about 1.7 times higher than that in women. In the same year, 73.09% (181,557[95% UI: 171,845–190,879]) of the deaths occurred in men, compared with 26.91% (66,852[95%UI: 62,553–71,159]) in women in East Asia and Pacific. The ASDR in men was about 2.1 times higher than that in women.

**Table 2:** The age-standardized rate and number of esophageal cancer cases in the world, East Asia and Pacific, China, Japan, and South Korea in 2017, by sex

Variables	World		East Asia and Pacific		China		Japan		South Korea	
	Male	Female	Male	Female	Male	Female	Male	Female	Male	Female
Incidence rate (per 100,000)	8.87 (8.55–9.16)	3.32 (3.17–3.47)	13.78 (13.05–14.51)	5.16 (4.79–5.51)	17.40 (16.30–18.50)	7.41 (6.80–8.01)	10.74 (10.12–11.34)	1.60 (1.50–1.70)	6.72 (5.79–7.76)	0.53 (0.46–0.60)
Incidence numbers	330,904 (319,159–341,987)	141,621 (135,089–147,968)	197,115 (186,163–207,760)	81,957 (76,083–87,598)	162,467 (152,103–172,885)	72,156 (66,266–78,056)	16,026 (15,074–16,918)	3,176 (3,000–3,350)	2,551 (2,174–2,962)	250 (218–285)
Death rate (per 100,000)	8.43 (8.15–8.70)	2.93 (2.81–3.05)	12.86 (12.19–13.52)	4.21 (3.94–4.48)	16.84 (15.83–17.83)	6.07 (5.61–6.53)	6.84 (6.54–7.15)	0.99 (0.95–1.04)	4.58 (4.03–5.15)	0.34 (0.31–0.38)
Death numbers	310,733 (300,244–320,551)	125,226 (120,125–130,421)	181,557 (171,845–190,879)	66,852 (62,553–71,159)	154,391 (144,950–163,636)	58,196 (53,819–62,609)	10,611 (10,140–11,098)	2,196 (2,097–2,294)	1,698 (1,491–1,913)	167 (149–187)
DALY rate (per 100,000)	184.27 (178.31–190.06)	60.96 (58.50–63.42)	266.85 (252.56–280.08)	78.05 (73.05–83.17)	341.20 (320.24–361.65)	107.60 (99.66–115.88)	141.11 (134.56–147.82)	20.43 (19.41–21.55)	89.47 (78.25–100.97)	6.28 (5.55–7.05)
DALY numbers	7,189,349 (6,956,383–7,415,160)	2,588,422 (2,483,936–2,693,257)	4,017,901 (3,801,341–4,219,275)	1,244,966 (1,165,297–1,327,324)	3,388,242 (3,176,038–3,594,450)	1,076,738 (997,302–1,160,383)	195,357 (186,788–204,307)	32,090 (30,489–33,731)	36,117 (31,521–40,880)	2,870 (2,535–3,214)

*DALY* disability-adjusted life-year

In 2017, 69.25% (162,467 [95% UI: 152,103–172,885]) of the incident cases occurred in men, compared with 30.75% (72,156 [95% UI: 66,266–78,056]) in women in China (Table 2). The ASIR of men was about 1.3 times higher than that of females. Of all deaths, 72.62% (154,391 [95% UI: 144,950–163,636]) occurred in men, compared with 27.38% (58,196 [95% UI: 53,819–62,609]) in women. The ASDR in men was about 1.7 times higher than that in women (Table 2).

Large disparities in ASIR and ASDR between the sexes were observed in 2017 for Japan and South Korea (ASIR in Japan: the male-to-female ratio was 6.5; ASDR in Japan: 6.9; ASIR in South Korea: 12.7; ASDR in South Korea: 13.5). Age-specific rates for both incidence and death increased with age; the same trend was seen in the world, East Asia and Pacific, and the three countries (Additional file 1: Figure S1). The burden of EC was rare in young people, and both death and incident rates peaked at the age of ≥ 80 years in both sexes.

### Trends in ASIR, ASDR, and age-standardized DALY rate

**Fig. 2:**
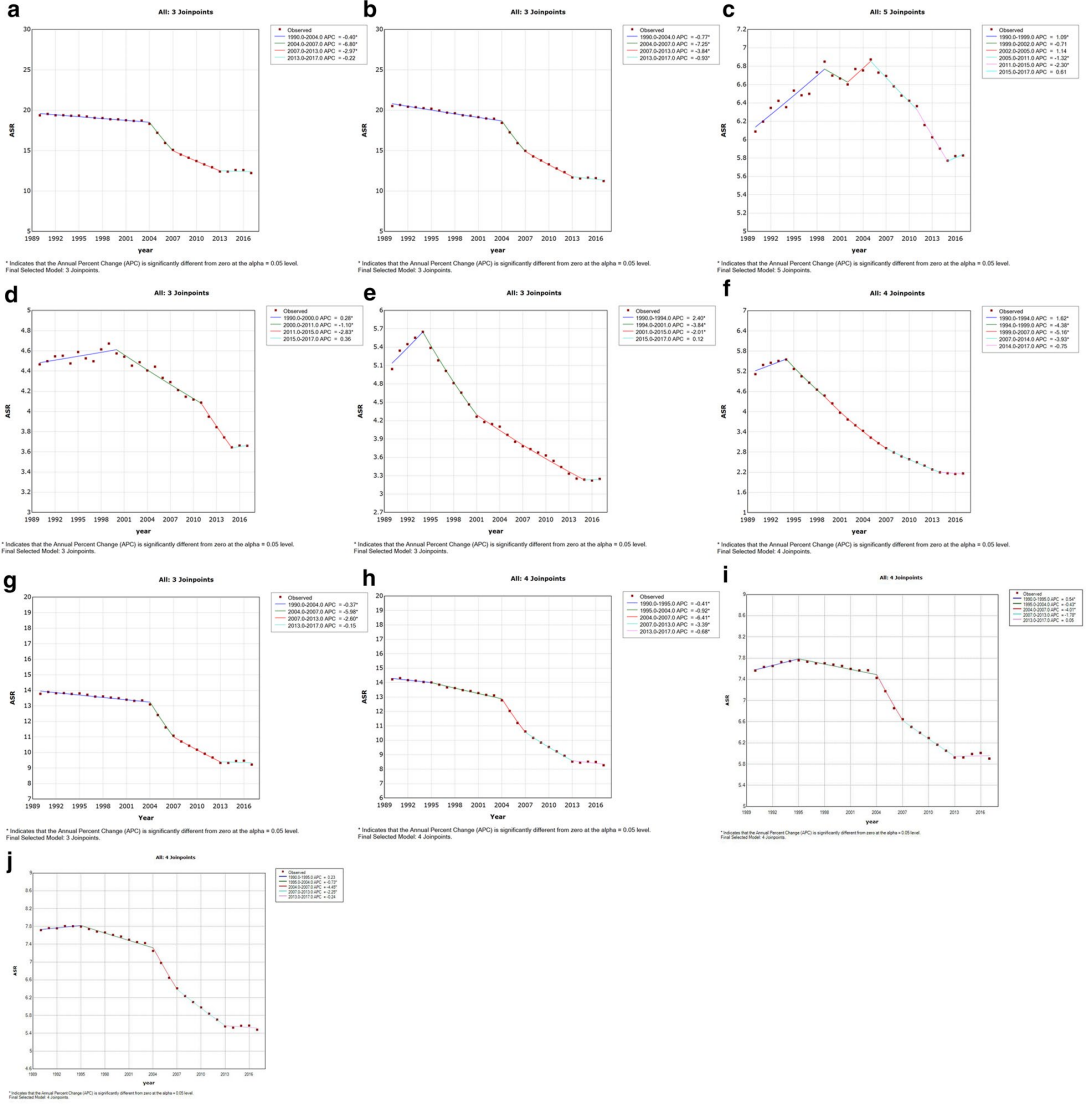
Trends for the ASIR and ASDR of esophageal cancer in the world, East Asia and Pacific, China, Japan, and South Korea from 1990 to 2017 calculated by joinpoint regression analyses. **a** ASIR in China; **b** ASDR in China; **c** ASIR in Japan; **d** ASDR in Japan; **e** ASIR in South Korea; **f** ASDR in South Korea; **g** ASIR in East Asia and Pacific; **h** ASDR in East Asia and Pacific; **i** ASIR in the world; **j** ASDR in the world. *ASIR* age-standardized incidence rate, *ASDR* age-standardized death rate

The results of the joinpoint regression analyses are shown in Fig. 2. In China, the ASIR declined in three periods (1990–2004: by 0.4%, *p* < 0.05, Fig. 2a; 2004–2007: by 6.80%, *p* < 0.05; 2007–2013: by 2.97%, *p* < 0.05). Similarly, the ASDR in China declined in four periods (1990–2004: by 0.77%, *p* < 0.05, Fig. 2b; 2004–2007: by 7.25%, *p* < 0.05; 2007–2013: by 3.84%, *p* < 0.05; 2013–2017: by 0.93%, *p* < 0.05).

Meanwhile, Japan showed different trends in ASIR and ASDR. ASIR first increased in one period and then declined in two periods (1990–1999: increased by 1.09%, *p* < 0.05, Fig. 2c; 2005–2011: declined by 1.32%, *p* < 0.05; 2011–2015: declined by 2.30%, *p* < 0.05). The first increasing and then decreasing trend in ASDR was found between 1990 and 2017 (1990–2000: increased by 0.28%, *p* < 0.05, Fig. 2d; 2000–2011: declined by 1.10%, *p* < 0.05; 2011–2015: declined by 2.83%, *p* < 0.05).The trends in ASIR and ASDR in South Korea were similar to the trends in Japan (ASIR: 1990–1994: increased by 2.40%, *p* < 0.05, Fig. 2e; 1994–2001: declined by 3.84%, *p* < 0.05; 2001–2015: declined by 2.01%, *p* < 0.05; ASDR: 1990–1994: increased by 1.62%, *p* < 0.05, Fig. 2f; 1994–1999: declined by 4.38%, *p* < 0.05; 1999–2007: declined by 5.16%, *p* < 0.05; 2007–2014: declined by 3.93%, *p* < 0.05). The trends in age-standardized DALY rate in China, Japan, and South Korea are displayed in Additional file 2: Figure S2.

### The associations between ASIR, ASDR, age-standardized DALY rate, and SDI in the world, China, Japan, and South Korea

**Fig. 3:**
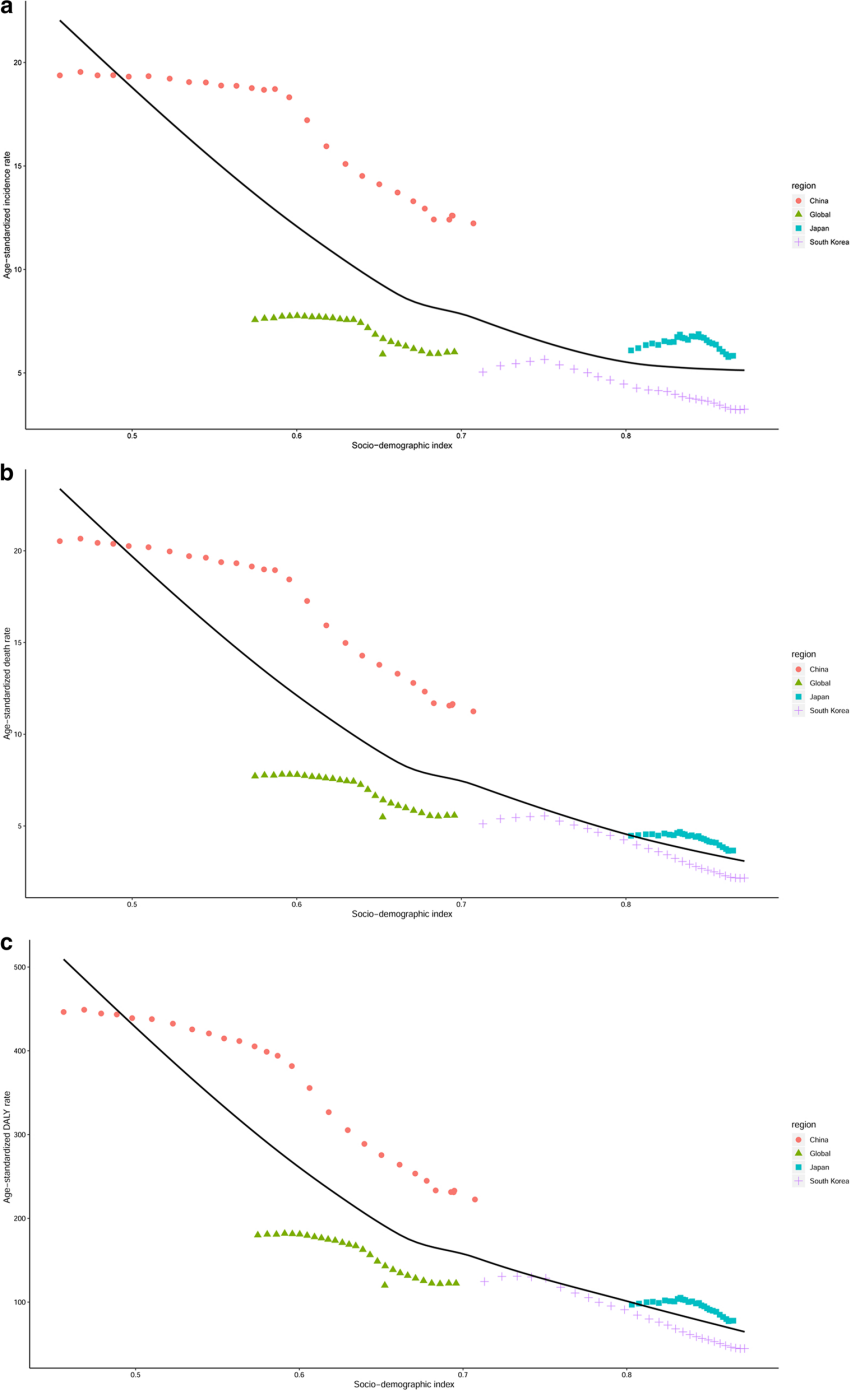
The trend in ASIR (**a**), ASDR (**b**), and age-standardized DALY rate (**c**) of esophageal cancer in the world, China, Japan, and South Korea by socio-demographic index for both sexes combined, 1990 to 2017. Expected values are shown as the black line. *ASIR* age-standardized incidence rate, *ASDR* age-standardized death rate, *DALY* disability-adjusted life-year

The association between ASIR, ASDR, age-standardized DALY rate, and SDI in the world, China, Japan, and South Korea from 1990 and 2017 are shown in Fig. 3. The trends in ASIR, ASDR, and age-standardized DALY rate showed similarities. Generally, ASRs in all regions presented a decreasing trend as the SDI value increased. Although all ASRs in China declined markedly with the increase in SDI value, they were above the expected levels in all years from 1990 to 2017. Interestingly, ASRs in Japan increased first and then decreased, and all ASRs remained above the expected levels in the past 28 years. As for South Korea, all ASRs were near the expected levels.

### EC burden in different areas in China

Huge geographic variations existed in ASIR and ASDR of EC among rural–urban residences and across the 34 provinces. Urban areas showed lower ASIR and ASDR (7.70 and 5.90 per 100,000 persons, respectively) than their rural counterparts (16.12 and 11.67 per 100,000 persons, respectively). Further, middle areas presented the highest ASIR and ASDR (12.75 and 9.30 per 100,000 persons, respectively). The lowest ASIR and ASDR (9.99 and 7.53 per 100,000 persons, respectively) were reported in eastern areas (Additional file 5: Table S1).

**Fig. 4:**
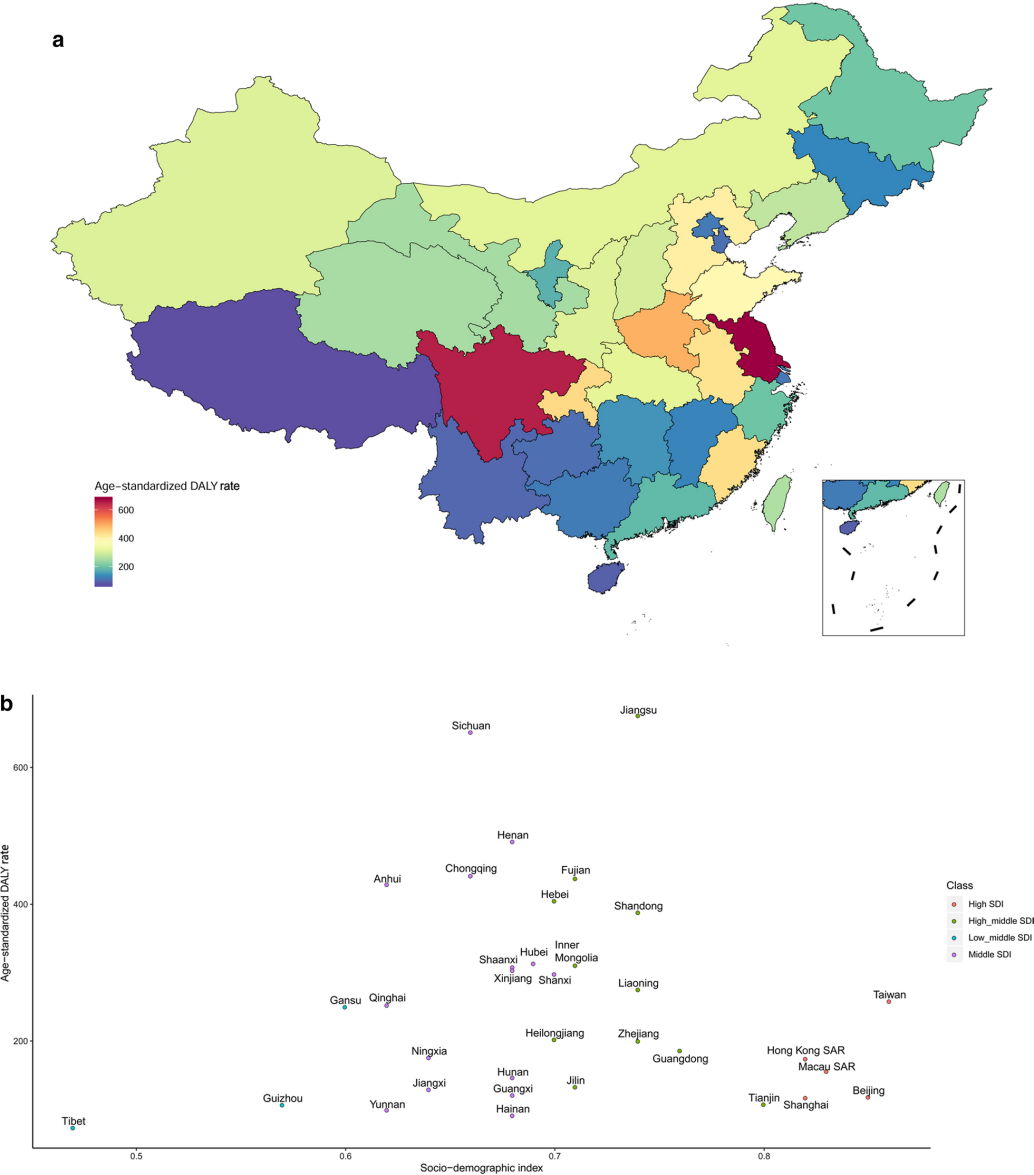
**a** The age-standardized DALY rate of esophageal cancer in the 34 provinces of China in 2017. **b** Age-standardized DALY rate and socio-demographic index in the 34 provinces of China in 2017. *DALY* disability-adjusted life-year

Table 3 shows the DALYs and DALY rates of EC in the 34 provinces of China from 1990 to 2017. In 2017, the three provinces with the greatest age-standardized DALY rate were Jiangsu (676.15[95% UI: 561.88–801.22]), Sichuan (651.79 [95% UI: 517.97–786.13]), and Henan (491.86 [95% UI: 412.39–579.31]). Similar to the pattern of ASIR and ASDR, the age-standardized DALY rate of EC decreased from middle areas to eastern areas (Fig. 4a). The age-standardized DALY rates and SDI values in the 34 provinces of China in 2017 are presented in Fig. 4b.

**Table 3:** DALY number and DALY rate from 1990 to 2017 for esophageal cancer in the 34 provinces of China

Location name	1990		2017	
	DALY number (in thousands)	DALY rate (per 100,000)	DALY number (in thousands)	DALY rate (per 100,000)
Taiwan	20.60 (19.90–21.30)	100.84 (97.57–104.41)	60.9 (55.9–66.4)	258.45 (237.20–281.47)
Hong Kong SAR	13.70 (12.30–15.30)	237.41 (213.36–266.16)	13.1 (10.8–16.0)	174.22 (143.59–212.80)
Macau SAR	0.52 (0.47–0.58)	147.32 (133.12–163.52)	1.07 (0.86–1.29)	156.09 (125.26–188.19)
Anhui	296 (258–339)	499.13 (435.82–571.63)	262 (220–310)	429.28 (359.06–507.10)
Beijing	21.5 (18.2–25.1)	191.80 (162.32–223.77)	28.6 (22.7–35.1)	118.47 (94.16–145.56)
Chongqing	71.4 (61–84.3)	452.71 (386.32–534.06)	118 (94.5–147)	441.82 (353.68–551.18)
Fujian	132 (118–151)	418.79 (371.49–477.83)	173 (142–211)	437.82 (359.23–534.46)
Gansu	82.5 (72.4–94.7)	348.93 (306.16–400.35)	66.5 (55.2–79.1)	250.26 (207.61–297.75)
Guangdong	173 (147–205)	263.28 (222.76–311.34)	218 (178–265)	186.24 (152.68–226.75)
Guangxi	50.6 (42.8–60.5)	116.25 (98.44–139.08)	58.5 (48.4–70.9)	121.15 (100.10–146.72)
Guizhou	31.4 (26.3–37.5)	94.74 (79.32–112.84)	36.2 (29.6–43.3)	106.99 (87.50–128.04)
Hainan	8.25 (6.89–9.81)	118.41 (98.85–140.90)	8.77 (6.87–11.20)	91.49 (71.61–116.72)
Hebei	418 (376–461)	656.86 (590.27–724.88)	310 (257–368)	405.15 (336.48–481.29)
Heilongjiang	48.7 (42.5–55.4)	133.68 (116.57–151.93)	79.5 (63.9–99.8)	202.49 (162.73–254.17)
Henan	695 (633–764)	787.16 (717.65–865.36)	474 (397–558)	491.86 (412.39–579.31)
Hubei	122 (109–135)	218.83 (196.87–242.87)	173 (145–208)	313.65 (262.87–375.23)
Hunan	69.3 (61.9–78)	111.40 (99.48–125.38)	98.6 (81.3–119)	146.88 (121.04–177.14)
Inner Mongolia	64.9 (55–76)	285.55 (241.78–334.12)	80.5 (66.5–98.1)	310.88 (256.63–378.71)
Jiangsu	318 (282–358)	462.56 (410.37–520.59)	552 (459–654)	676.15 (561.88–801.22)
Jiangxi	80.5 (71–91.1)	203.60 (179.53–230.47)	61.5 (52.3–72.6)	129.44 (109.99–152.74)
Jilin	28.9 (25.3–33.1)	111.44 (97.41–127.62)	37.2 (29.1–45.4)	133.21 (104.16–162.89)
Liaoning	78.2 (64.7–93.4)	191.66 (158.54–229.06)	123 (98.1–152)	275.46 (219.61–340.01)
Ningxia	9.31 (7.99–10.90)	188.43 (161.75–220.91)	12.4 (9.86–15.6)	176.06 (140.52–221.60)
Qinghai	11.7 (10–13.8)	246.69 (211.34–290.52)	15.9 (12.7–19.2)	252.61 (202.47–305.25)
Shaanxi	65.8 (57.5–74.5)	191.15 (167.15–216.46)	122 (97.4–152)	308.25 (246.48–384.45)
Shandong	351 (305–406)	410.34 (356.69–473.98)	386 (318–464)	388.29 (320.16–466.73)
Shanghai	30.5 (26.4–35.6)	218.62 (188.78–254.60)	32.7 (26.3–40.2)	117.21 (93.98–143.92)
Shanxi	144 (126–164)	475.81 (416.37–541.29)	115 (92.3–140)	298.14 (238.84–363.70)
Sichuan	467 (415–537)	418.43 (371.91–480.77)	544 (432–656)	651.79 (517.97–786.13)
Tianjin	16.1 (13.8–18.4)	175.51 (150.51–200.17)	16.4 (13.5–20)	107.68 (88.45–130.90)
Tibet	3.22 (2.76–3.76)	138.39 (118.63–161.24)	2.54 (2.09–3.05)	73.55 (60.47–88.27)
Xinjiang	42.5 (37.7–47.4)	269.59 (239.01–300.57)	74 (61.8–88.8)	303.72 (253.42–364.52)
Yunnan	34.5 (28.7–42.6)	88.63 (73.60–109.21)	48.9 (40.3–60.2)	99.49 (81.95–122.42)
Zhejiang	97.4 (84.6–113)	220.93 (191.83–256.76)	122 (97.7–151)	200.15 (159.64–246.67)

*DALY* disability-adjusted life-year, *SAR* special administrative region

### Risk factors attributable to EC death and DALYs in the world, East Asia and Pacific, China, Japan, and South Korea

Generally, tobacco was the greatest contributor to death and DALYs (including all-age and age-standardized) in the world, East Asia and Pacific, China, Japan, and South Korea (Additional file 3, 4: Figures S3 and S4). We found differences in trends in the percentages of death and DALYs from tobacco use in the three countries from 1990 to 2017. Worldwide tobacco-related DALYs and death (including all-age and age-standardized) remained stable between 1990 and 2017. However, China showed markedly increasing trends in tobacco-related DALYs and death, whereas Japan exhibited markedly declining trends, and South Korea showed an increasing trend and then a decreasing one. Moreover, the percentage of high BMI-related DALYs and death increased and that of low-fruit diet decreased in these regions over time.

**Fig. 5:**
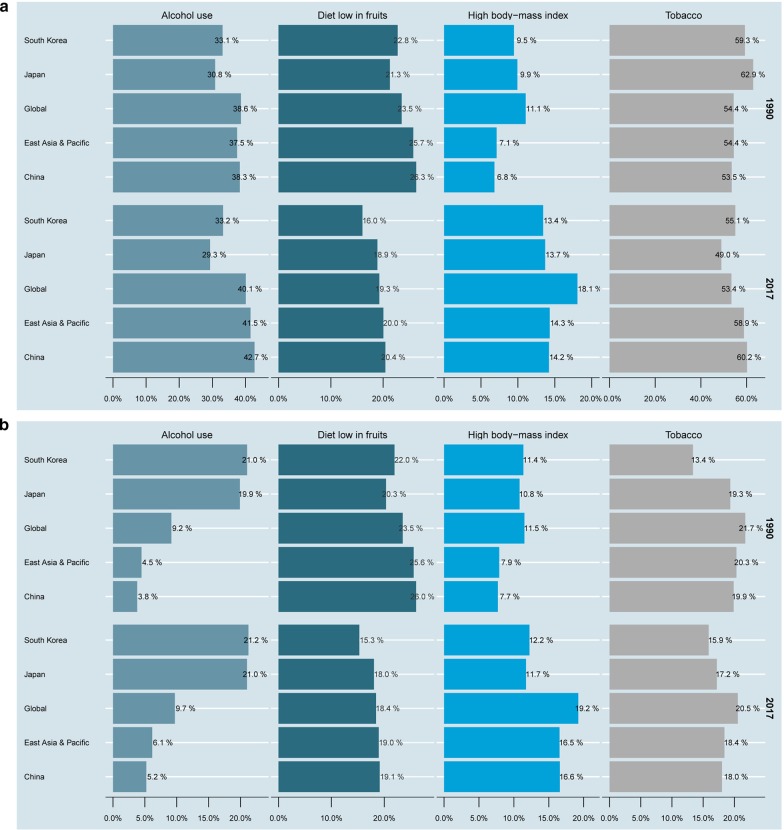
Percentage of esophageal cancer age-standardized death due to tobacco, alcohol use, high body-mass index, and diet low in fruits in the world, East Asia and Pacific, China, Japan, and South Korea in 1990 and 2017. **a** For men; **b** for women

The percentage of EC age-standardized deaths (ASDs) owing to risk factors differed across countries and sexes in 2017 (Fig. 5a, b). Specifically, the percentage of ASDs owing to tobacco for men was the highest in China (60.2%), well above the level of East Asia and Pacific (58.9%) and the global level (53.4%), and the lowest in Japan (49.0%). The highest percentage of ASDs owing to alcohol use for men was found in China (42.7%), likewise above the level of East Asia and Pacific (41.5%) and the global level (40.1%), and the lowest in Japan (29.3%). China also showed the highest percentage of ASDs owing to high BMI (14.2%), which was below the level of East Asia and Pacific (14.3%) and the global level (18.1%). The lowest percentage of ASDs owing to high BMI for men was found in South Korea (13.4%). As for the percentage of ASDs owing to low-fruit diet for men, the lowest was found in South Korea (16.0%) and the highest in China (20.4%), above the level of East Asia and Pacific (20.0%) and the global level (19.3%).

In 2017, the percentage of ASDs owing to tobacco and low-fruit diet for women was the highest in China (18.0% and 19.1%, respectively) and the lowest in South Korea (15.9% and 15.3%, respectively). South Korea (21.2%) and Japan (21.0%) had the highest percentage of ASDs owing to alcohol use for women, exceeding the level of East Asia and Pacific (6.1%) and the global level (9.7%); China had the lowest percentage (5.2%). As for the percentage of ASDs owing to high BMI for women, the lowest was found in Japan (11.7%) and the highest in China (16.6%). In summary, tobacco and alcohol use were the most important risk factors for ASDs in men in three countries and in women in Japan and South Korea. Meanwhile, in Chinese women, high BMI and low-fruit diet were the risk factors that contributed most to ASDs.

**Fig. 6:**
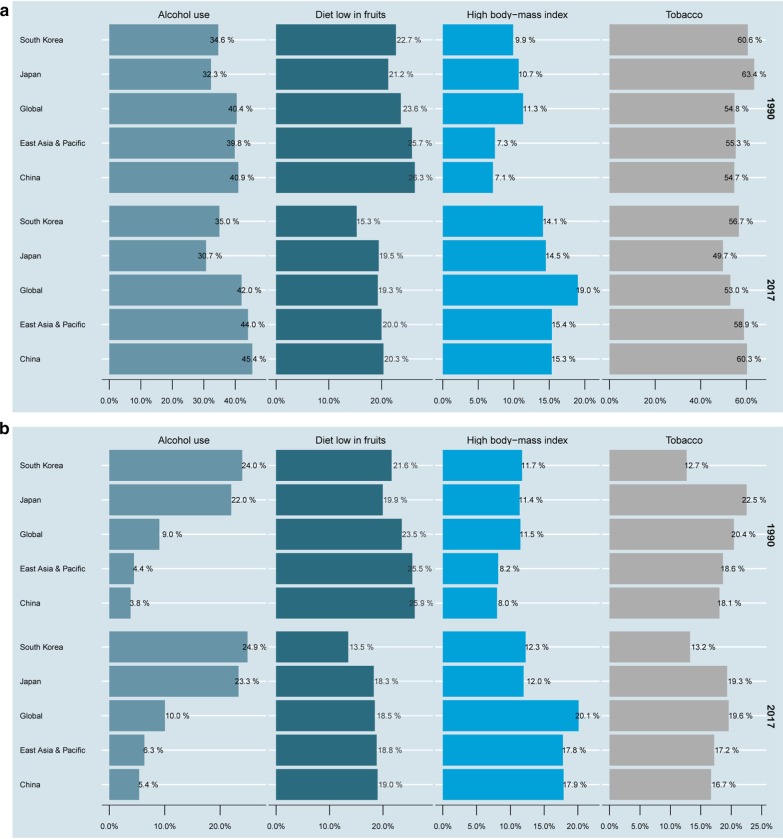
Percentage of esophageal cancer age-standardized DALY rate due to tobacco, alcohol use, high body-mass index, and diet low in fruits in the world, East Asia and Pacific, China, Japan, and South Korea in 1990 and 2017. **a** For men; **b** for women. *DALY* disability-adjusted life-year

The percentages of EC age-standardized DALY rate owing to risk factors also differed across countries and gender sexes in 2017(Fig. 6a, b). Tobacco and alcohol use were the most important risk factors in men in the three countries and in women in Japan and South Korea. Meanwhile, high BMI and low-fruit diet were the risk factors that contributed the most in Chinese women.

## Discussion

The burden and trends of EC in the world, East Asia and Pacific, China, Japan, and South Korea between 1990 and 2017 were systemically described in the present study. The incident and death cases of EC in the world, East Asia and Pacific, and the three countries increased from 1990 to 2017, whereas ASIR, ASDR, and age-standardized DALY rate decreased. In addition, we found that the incident and death rates of EC increased with age, especially in people aged ≥ 45 years, peaking at the seventh and eighth decades of life in the world, East Asia and Pacific, China, Japan, and South Korea. As populations age worldwide, EC will create a heavier burden on public health care systems.

Our results confirmed that men carried the majority of the incident and death rates of EC [1, 21]. However, the reason for sex disparities had not been clarified. Smoking and heavy alcohol consumption have been shown to increase the risk of EC [22, 23]. Historically, these behaviors have been more common in men than in women [24]. This might partly explain the higher burden of EC in men than in women. Our study verified that the percentages of ASDs and DALYs owing to tobacco and alcohol use were higher in men than in women.

The regional variations in the ASIR and ASDR of EC for both sexes are huge across China, Japan, and South Korea, which may be explained by the heterogeneity in the prevalence of risk factors. Socioeconomic status is a risk factor for EC. In our study, we used SDI to reflect socioeconomic status. All ASRs decreased as the SDI increased. However, the association between socioeconomic status (SDI) and the ASIR, ASDR, and age-standardized DALY rate is complex and nonlinear. The declines we found might be from increasing socioeconomic levels and dietary improvements. Although the ASIR and ASDR in China declined with SDI value, they were above the expected levels in the past 28 years and may be attributed to the behavioral and dietary risk factors of EC and lack of early diagnosis and monitoring methods. Meanwhile, the ASIR in South Korea was much lower than the expected levels, which, we speculate, can be attributed to the prevention of the other risk factors of EC in South Korea.

Our study revealed that China, Japan, and South Korea had different risk factor profiles of EC. A 2005 report attributed 46% of the incidence of and death from EC in China to smoking, alcohol drinking, and low intake of vegetables and fruits [25]. Tobacco smoking remains the key risk factor for EC. Indeed, smoking reportedly accounts for a larger percentage of risk in developed regions or countries [26], whereas relatively lower attributable risk has been observed in less developed regions or countries [27, 28]. In contrast, our study revealed that the percentages of ASDs and DALYs owing to tobacco were higher in China (less developed country) than in Japan and South Korea (developed countries). Further, China showed significantly increased trends in tobacco-related DALYs and deaths over the 28-year study period. Many studies have revealed that alcohol use is a risk factor for EC in both developing and developed regions. Alcohol use increases the risk of EC 1.6- to 5.3-fold in Asian countries [12, 29] and about threefold in African [30] and South American countries [31]. The association between alcohol use and EC appears to be stronger in regions with low burden of EC, such as Europe [32, 33]. Our study showed that the high percentage of ASDs owing to alcohol use existed in Chinese men and in South Korean and Japanese women. As for BMI, an increase of 5 kg/m^2^ enhances the risk of EC by approximately 10% [34]. A systematic review reported that high intake of fruits can reduce the risk and morbidity of EC [35]. Our study identified high BMI and low-fruit diet as the two risk factors that contributed largely to ASDs in Chinese women. However, the trends in deaths and DALYs owing to these two risk factors were different. Deaths and DALYs associated with high BMI showed an increasing trend, whereas the percentage of deaths and DALYs associated with low-fruit diet had been declining in China in the past 28 years. China has the largest number of obesity and overweight people, attributed to changes in food environment and systems [36]. In all, strategies for health promotion should take tobacco, alcohol use, and high BMI into consideration. Patients exposed to these risk factors should be provided with proper prevention and education. In China, quitting smoking, reducing alcohol consumption, and reducing weight should be essential components of prevention strategies.

Our study has some limitations. First, the study lacked information on the histological subtypes of EC. The epidemiological features of ESCC and EAC appear to differ substantially. However, owing to a lack of necessary data, we could not identify the burden and risk factors in these two histological subtypes. Second, data on the type of risk factors in this study were limited. More factors that might be attributable to EC, such as *Helicobacter pylori* infection, should be investigated in future studies.

## Conclusions

Incident cases and deaths in China, Japan, and South Korea increased from 1990 to 2017, whereas the ASIR, ASDR, and age-standardized DALY rate declined. With an aging population, EC will create a heavier burden on public health care systems. Risk factors, including smoking, alcohol use, high BMI, and low-fruit diet, are the main factors of death and DALYs and should thus be given importance.

## Supplementary information

Additional file 1: Figure S1. Age-specific rates for incidence, death, and DALY increased with age. DALY: disability-adjusted life-year.

Additional file 2: Figure S2. Trends for age-standardized DALY rate of esophageal cancer in China (A), Japan (B), South Korea (C), East Asia and Pacific (D), and the world (E) from 1990 to 2017 calculated by joinpoint regression analyses. DALY: disability-adjusted life-year.

Additional file 3: Figure S3. Trends in percentage of esophageal cancer all-age death and ASDR due to risk factors in the world, East Asia and Pacific, China, Japan, and South Korea, from 1990 to 2017. (A) all-age death; (B) ASDR. ASDR: age-standardized death rate.

Additional file 4: Figure S4. Trends in percentage of esophageal cancer all-age and age-standardized DALY rate due to risk factors in the world, East Asia and Pacific, China, Japan, and South Korea, from 1990 to 2017. (A) all-age DALYs; (B) age-standardized DALY rate. DALY: disability-adjusted life-year.

**Additional file 5:** Table S1. Incident cases, death cases, age-standardized incidence rate (ASIR), and age-standardized death rate (ASDR) for esophageal cancer in China, by geographic areas, 2015.

## Data Availability

The datasets supporting the conclusions of this article are included within the article.

## Abbreviations

EC: Esophageal cancer
DALY: Disability-adjusted life-year
ASIR: Age-standardized incidence rate
ASDR: Age-standardized death rate
SDI: Socio-demographic index
BMI: Body mass index
ESCC: Esophageal squamous cell carcinoma
EAC: Esophageal adenocarcinoma
SAR: Special Administrative Region
UI: Uncertainty interval
ASR: Age-standardized rate
ASD: Age-standardized death
