# Supplementary figures and images for: Burden, trends, and risk factors of esophageal cancer in China from 1990 to 2017: an up-to-date overview and comparison with those in Japan and South Korea
Source: J Hematol Oncol. 2020 Nov 2;13:146. doi: 10.1186/s13045-020-00981-4 (PMC7607864; doi:10.1186/s13045-020-00981-4)

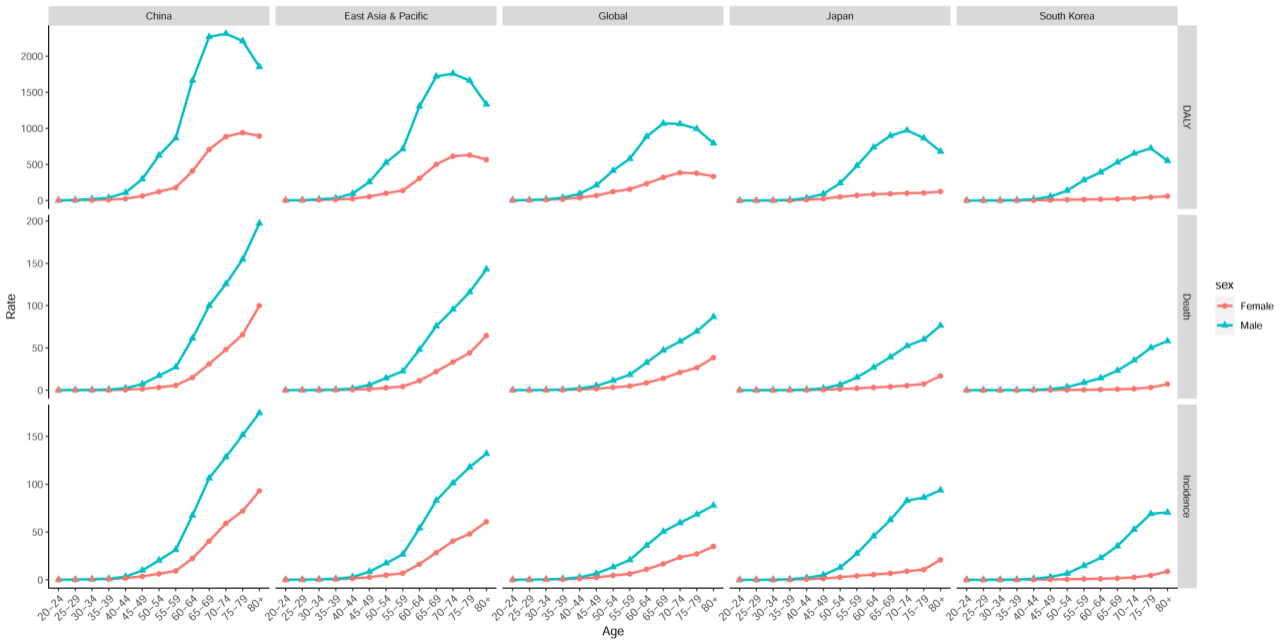

Supplement: Supplementary file 1 — Figure S1. Age-specific rates for incidence, death, and DALY increased with age. DALY: disability-adjusted life-year. [file 13045_2020_981_MOESM1_ESM.pdf]

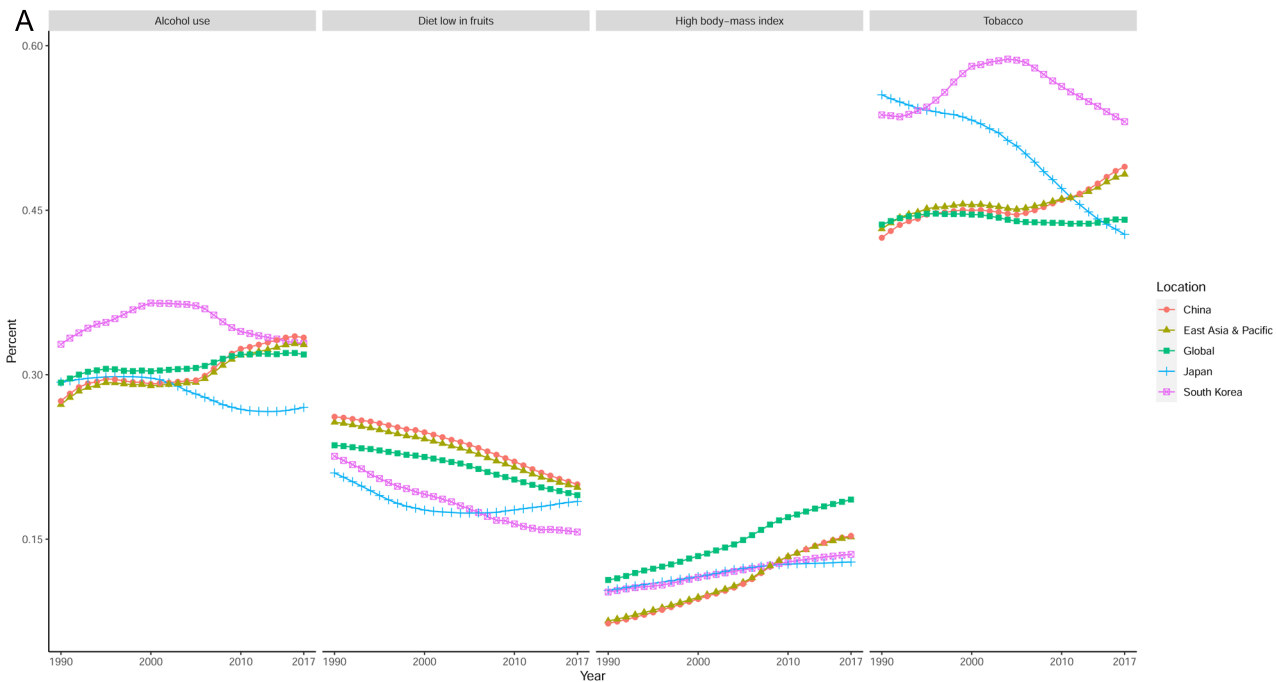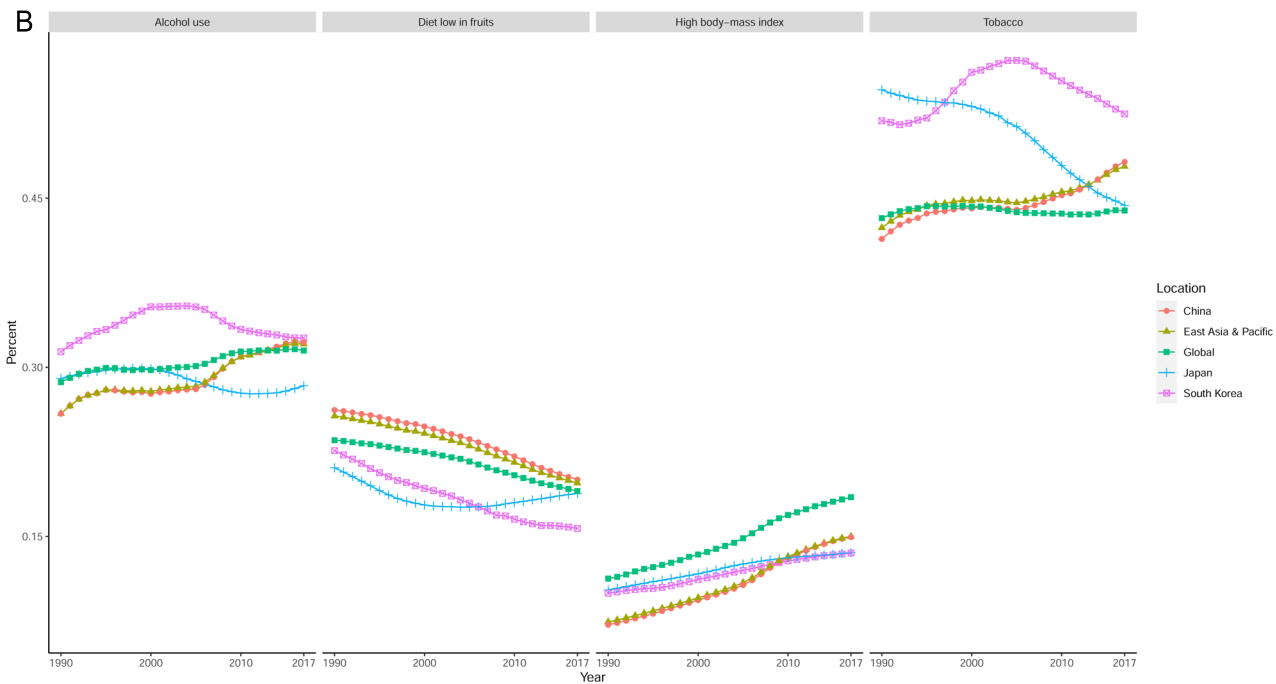

Supplement: Supplementary file 3 — Figure S3. Trends in percentage of esophageal cancer all-age death and ASDR due to risk factors in the world, East Asia and Pacific, China, Japan, and South Korea, from 1990 to 2017. (A) all-age death; (B) ASDR. ASDR: age-standardized death rate. [file 13045_2020_981_MOESM3_ESM.pdf]

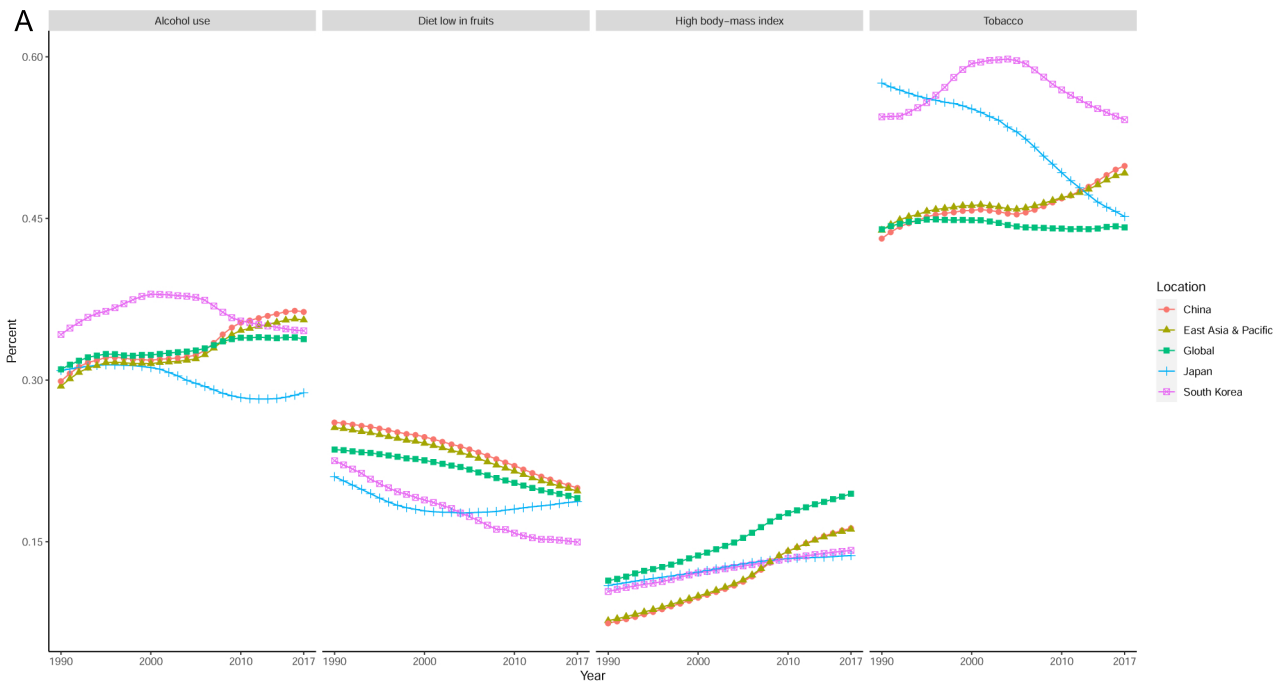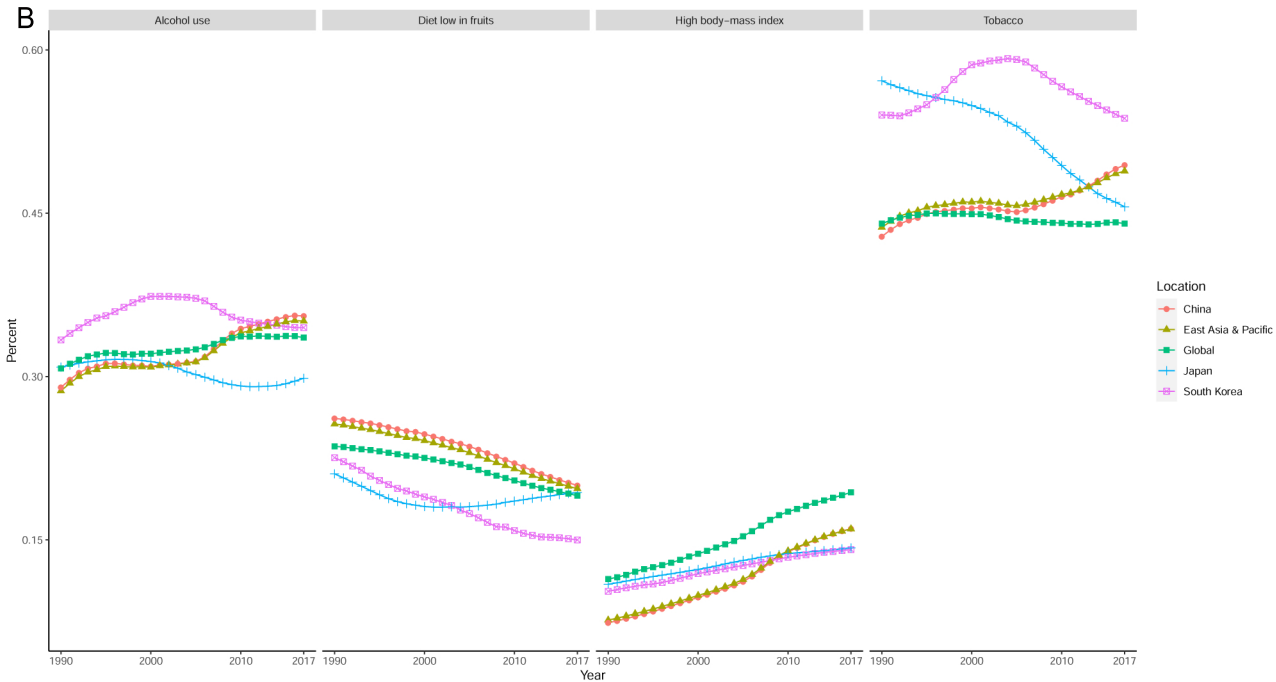

Supplement: Supplementary file 4 — Figure S4. Trends in percentage of esophageal cancer all-age and age-standardized DALY rate due to risk factors in the world, East Asia and Pacific, China, Japan, and South Korea, from 1990 to 2017. (A) all-age DALYs; (B) age-standardized DALY rate. DALY: disability-adjusted life-year. [file 13045_2020_981_MOESM4_ESM.pdf]
