# Supplementary material for: Burden, trends, and risk factors of esophageal cancer in China from 1990 to 2017: an up-to-date overview and comparison with those in Japan and South Korea
Source: J Hematol Oncol. 2020 Nov 2;13:146. doi: 10.1186/s13045-020-00981-4 (PMC7607864; doi:10.1186/s13045-020-00981-4)
Supplement: Supplementary file 2 — Figure S2. Trends for age-standardized DALY rate of esophageal cancer in China (A), Japan (B), South Korea (C), East Asia and Pacific (D), and the world (E) from 1990 to 2017 calculated by joinpoint regression analyses. DALY: disability-adjusted life-year. [file 13045_2020_981_MOESM2_ESM.pdf]

A

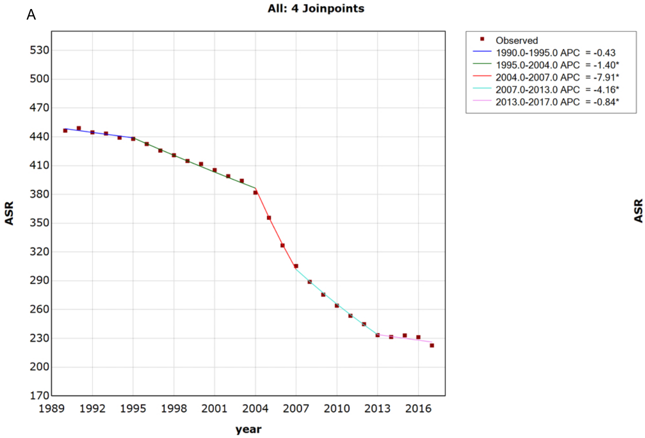

* Indicates that the Annual Percent Change (APC) is significantly different from zero at the alpha = 0.05 level.
Final Selected Model: 4 Joinpoints.

B

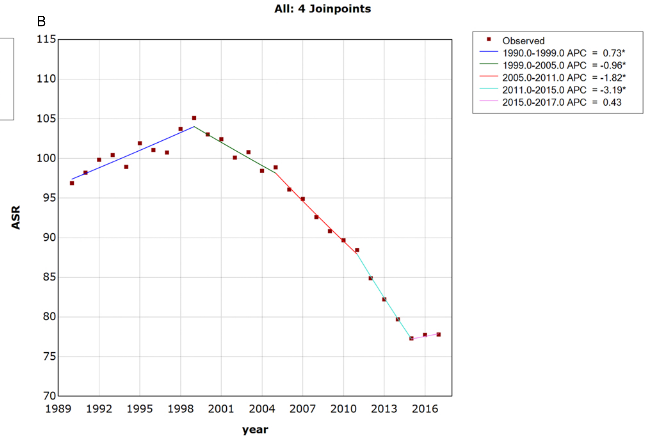

* Indicates that the Annual Percent Change (APC) is significantly different from zero at the alpha = 0.05 level.
Final Selected Model: 4 Joinpoints.

C

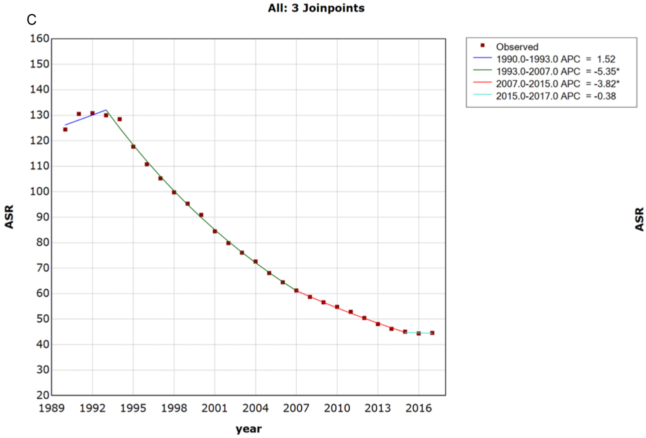

* Indicates that the Annual Percent Change (APC) is significantly different from zero at the alpha = 0.05 level.
Final Selected Model: 3 Joinpoints.

D

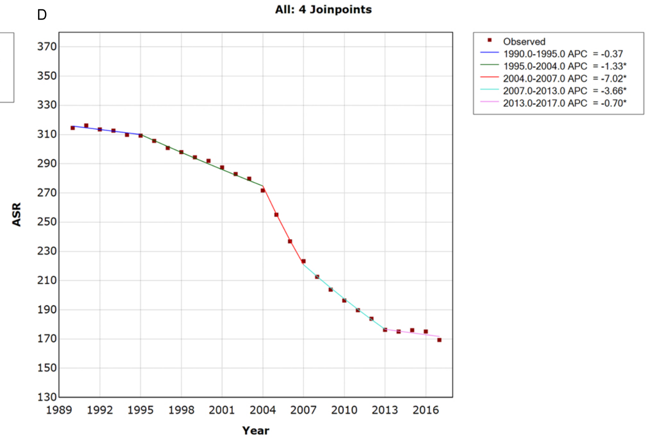

* Indicates that the Annual Percent Change (APC) is significantly different from zero at the alpha = 0.05 level.
Final Selected Model: 4 Joinpoints.

E

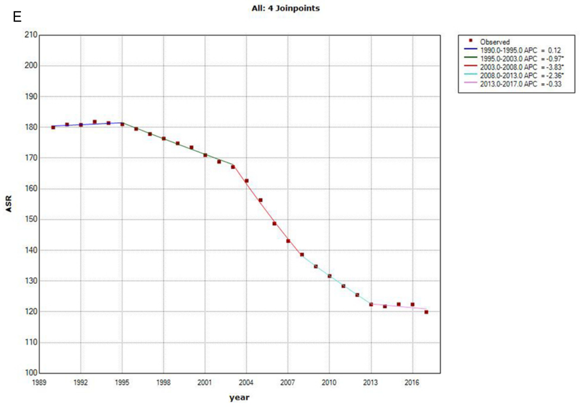

* Indicates that the Annual Percent Change (APC) is significantly different from zero at the alpha = 0.05 level.
Final Selected Model: 4 Joinpoints.
