# Supplementary material for: Burden, trends, and risk factors of esophageal cancer in China from 1990 to 2017: an up-to-date overview and comparison with those in Japan and South Korea
Source: J Hematol Oncol. 2020 Nov 2;13:146. doi: 10.1186/s13045-020-00981-4 (PMC7607864; doi:10.1186/s13045-020-00981-4)
Supplement: Supplementary file 5 — Additional file 5: Table S1. Incident cases, death cases, age-standardized incidence rate (ASIR), and age-standardized death rate (ASDR) for esophageal cancer in China, by geographic areas, 2015. [file 13045_2020_981_MOESM5_ESM.docx]

**Table S1. Incident cases, death cases, age-standardized incidence rate (ASIR), and age-standardized death rate (ASDR) for esophageal cancer in China, by geographic areas, 2015.**

| **Geographic areas** | **Sex** | **Incidence** | | | **Death** | | |
| --- | --- | --- | --- | --- | --- | --- | --- |
|  |  | **Cases(in thousands)** | **Rate(1/100000)** | **ASIR(1/100000)** | **Cases(in thousands)** | **Rate(1/100000)** | **ASDR(1/100000)** |
| **All areas** | Both | 246 | 17.87 | 11.28 | 188 | 13.68 | 8.36 |
|  | Male | 177 | 25.13 | 16.75 | 137 | 19.45 | 12.74 |
|  | Female | 69 | 10.25 | 5.94 | 51 | 7.62 | 4.14 |
| **Eastern areas** | Both | 89 | 17.18 | 9.99 | 69 | 13.40 | 7.53 |
|  | Male | 64 | 24.54 | 15.10 | 50 | 19.06 | 11.55 |
|  | Female | 24 | 9.60 | 5.03 | 19 | 7.57 | 3.69 |
| **Middle areas** | Both | 90 | 19.57 | 12.75 | 68 | 14.71 | 9.30 |
|  | Male | 63 | 26.33 | 18.19 | 48 | 20.08 | 13.64 |
|  | Female | 28 | 12.37 | 7.42 | 20 | 9.00 | 5.15 |
| **Western areas** | Both | 67 | 16.80 | 11.46 | 51 | 12.85 | 8.48 |
|  | Male | 50 | 24.49 | 17.47 | 39 | 19.23 | 13.45 |
|  | Female | 17 | 8.62 | 5.50 | 12 | 6.07 | 3.62 |

ASIR=Age-standardized incidence rate; ASDR= Age-standardized death rate.
